# Optimization of Lipoplexes Functionalized with a Sialic Acid Mimetic (F9-PEG) to Target the C1858T *PTPN22* Variant for Preclinical Assessment of a Novel Immunotherapy in Endocrine Autoimmunity

**DOI:** 10.3390/pharmaceutics17060710

**Published:** 2025-05-28

**Authors:** Simona Sennato, Giorgia Paldino, Cecilia Bombelli, Irene Mezzani, Stefania Petrini, Eugenia Belcastro, Domenico Vittorio Delfino, Francesco Fiorentino, Carlotta Marianecci, Alessia Ciogli, Dante Rotili, Francesca Ceccacci, Alessandra Fierabracci

**Affiliations:** 1 CNR-Institute for Complex Systems (ISC), and Physics Department, Sapienza University of Rome, P. le A. Moro 5, 00185 Rome, Italy; simona.sennato@cnr.it; 2 Bambino Gesù Children’s Hospital, Istituto di Ricovero e Cura a Carattere Scientifico (IRCCS), 00146 Rome, Italy; giorgiapld@gmail.com (G.P.); irene.mezzani@opbg.net (I.M.); stefania.petrini@opbg.net (S.P.); eugenia.belcastro@unipi.it (E.B.); 3 CNR-Institute for Biological Systems, Secondary Office of Rome—Sapienza University of Rome, 00185 Rome, Italy; cecilia.bombelli@cnr.it; 4 Department of Medicine and Surgery, University of Perugia, 06129 Perugia, Italy; domenico.delfino@unipg.it; 5 Department of Drug Chemistry and Technologies, Sapienza University of Rome, 00185 Rome, Italy; f.fiorentino@uniroma1.it (F.F.); carlotta.marianecci@uniroma1.it (C.M.); alessia.ciogli@uniroma1.it (A.C.); 6 Department of Science, Roma Tre University, 00146 Rome, Italy; dante.rotili@uniroma3.it

**Keywords:** type 1 diabetes, endocrine autoimmunity, immunotherapy, variant *PTPN22*, lipoplexes optimization

## Abstract

**Background:** The C1858T *PTPN22* variant is strongly associated with type 1 diabetes and autoimmune thyroid disease. Current treatment is substitutive hormonal administration, which does not target the disease pathogenetic mechanism. We previously implemented a novel immunotherapy, employing siRNA directed against the C1858T variant of *PTPN22* delivered via functionalized lipoplexes, in order to halt autoimmune disease progression. **Objectives**: The objective of this study was to optimize lipoplex formulations functionalized with F9-PEG (a Siglec-10’s ligand) to facilitate targeted delivery by investigating their physical and chemical properties to warrant the best performance in in vivo studies. **Methods**: The effectiveness of siRNA liposome binding was evaluated by varying the relative lipid/siRNA charge ratio and analyzing the stability of the different formulations with respect to the methods of F9-PEG addition and ATTO740 fluorescent labeling by electrophoresis, dynamic and dielectrophoretic light scattering (DLS and DELS), and high-performance liquid chromatography (HPLC). **Results**: The optimal charge ratio of +2/−1 (lipid/siRNA) ensured a greater stability of lipoplexes and complete complexation of siRNA. Stability was improved by selecting a protocol of preparation that envisages functionalization with F9-PEG and the addition of ATTO740 lipid in the lipid film preparation step. HPLC confirmed the integrity of siRNA after preparation. **Conclusions**: The results of this study lead to formulations of F9-PEG lipoplexes with optimal properties that could be used for biodistribution and safety/efficacy studies in mice. Lipoplexes functionalized with F9-PEG could therefore represent a promising personalized nanotherapeutic platform for targeted siRNA delivery in endocrine C1858T patients.

## 1. Introduction

Autoimmune thyroid disease (ATD) and insulin-dependent diabetes mellitus (type 1 diabetes, T1D) are caused by the immune attack of thyrocytes and beta cells, respectively, by autoreactive T cells [1]. The combination is known as autoimmune polyglandular syndrome Type 3 variant (APS3v) [2].

The global annual rate of T1D incidence is rising, particularly below the age of 5 years, frequently presenting with ATD [3]. The current treatment is substitutive hormone administration, which has no effect on the pathogenetic autoimmune mechanism and does not preserve the residual hormonal cells [4]. Identification of innovative immunotherapies, especially aimed at preserving the residual cells, is therefore of upmost relevance for the quality of life in patients affected since pediatric age [5]. A strong genetic background underlies the pathogenesis of these endocrine autoimmune conditions [2], with several gene variants discovered. Among these, particular attention is paid to the potential pathophysiological role played in autoimmune disorders, including T1D and APS3v, by the C1858T variant of the *PTPN22* (protein tyrosine phosphatase N22) gene [6], which encodes for the R620W variant Lyp protein tyrosine phosphatase [6].

Lyp, through its interaction with C-terminal Src kinase (CSK), is a negative regulator of T cell antigen receptor (TCR) signaling. The Lyp R620W variant leads to a gain of function mutation with paradoxical reduced T cell activation [7]. Peripheral blood T lymphocytes (PBMC) of T1D patients are indeed hyporesponsive to in vitro anti-CD3 [aCD3]. Subtle TCR signaling defects induced by the Lyp variant could influence central thymus tolerance and the escape of autoreactive T cells [7]. The variant *PTPN22* has effects on innate and adaptive immunity. We also observed altered B cell homeostasis and response to CpG stimulation in C1858T T1D patients [8]. In light of this, LypR620W may be a valid drug target for T1D immunotherapy based on gene silencing. In this regard, siRNAs (small interfering RNAs) constituted by two antisense strands intended to recognize a target RNA have proven to be a more robust technology [9] than antisense oligonucleotides (ASO), ribozymes, DNAzymes, or RNAi. ASO systemic administration has already shown to be feasible in treating disease-related genes. However, with specific reference to immunotherapies, the utility of antisense technology already presents limitations not only for molecules’ high extent of degradation but also for their inefficient transport across the plasma membranes of immunocytes—in particular, T and B lymphocytes.

We implemented a novel immunotherapy based on the use of siRNA causing variant allele-selective inhibition instead of complete gene knockdown [10,11]. A fundamental aim in exploiting novel immunotherapies is the realization of efficient and safe delivery agents of siRNAs. Thus, in order to improve delivery, siRNAs were loaded into liposomes (lipoplexes). We already demonstrated the feasibility of the approach of using lipoplexes to obtain the inhibition of the C1858T allelic variant of *PTPN22* in T1D peripheral blood mononuclear cells (PBMCs) [12]. Furthermore, we implemented lipoplexes through functionalization to improve selective delivery to specific immunocytes; in particular, we explored the possibility to expose Fab from monoclonal antibodies targeting CD20 (Rituximab) to improve delivery to B lymphocytes [13]. Based on the experience carried out with Fab of Rituximab, siRNA lipoplexes will be improved by conjugation with aCD3 FDA approved mAbs to achieve specific silencing in cytotoxic T cells. An alternative strategy of functionalization to target several immunocytes in the peripheral blood was exploited using high affinity Siglec-10 sialoside mimetic (SAM, F9-PEG lipid) [14,15]. The Siglec family of sialic acid-binding proteins (Siglecs) are mainly expressed on immunocytes mediating innate and adaptive responses [15]. We previously demonstrated that PEG-F9 functionalization provides a more efficient inhibition of the variant *PTPN22* gene in PBMC of heterozygous T1D patients than the non-functionalized lipoplexes [16]. In view of the prospective use of the prepared and characterized lipoplexes for immunotherapy of T1D and APS3v, a fundamental step forward is to estimate the biodistribution of lipoplexes and of their functionalized derivatives by injections in C57BL/6 mice. It is also essential to assess their safety and efficacy in delaying or halting the disease’s development in the NOD mouse model of T1D, which harbors the R619W variant of *Ptpn22*, equivalent to the human R620W *PTPN22* variant.

In light of the foregoing, in this manuscript, we aimed to optimize the formulations of lipoplexes functionalized with F9-PEG, with a view toward ensuring optimal performance for future in vivo experiments. We explored different protocols of preparation to select the most effective to provide lipoplexes that are more stable over time, with a suitable diameter and PDI. We also investigated the effect of the fluorescent lipid probe ATTO740-DMPC (ATTO740) on the stability of the formulations prepared following the different protocols.

## 2. Materials and Methods

### 2.1. siRNA Design

Authentic siRNA sequences to silence the C1858T *PTPN22* gene variant (Rosetta Inpharmatics, Sigma-Aldrich Chemical Co., St. Louis, MO, USA) were reported [11] (Italian Patent 102018000005182 released on 26 June 2020; PCT/IT2019/050095 filed on 8 May 2019 in Europe, USA and China, Inventor: Dr. A. Fierabracci).

### 2.2. Liposome/Lipoplex Preparation and Characterization

#### 2.2.1. General Liposome and Lipoplex Preparations

Additional details are provided in previously published manuscripts [11,12,13] referring to protocols for liposome preparations. Briefly, gemini surfactant *2R,3S*-2,3-dimethoxy-1,4-bis (N-hexadecyl-N, N-dimethylammonium)-butane dibromide (gemini) was prepared following a reported procedure [17,18] (Figure S1). The aqueous dispersion of liposomes composed of DMPC (purity > 99%, Avanti Polar Lipids Inc. (Alabaster, AL, USA) and gemini was prepared according to the following established protocol [19]. A thin lipid layer was obtained inside the surface of a round-bottomed flask by evaporation of a CHCl_3_ solution containing DMPC and gemini at a 50/50 molar ratio. The film was dried overnight (O/N) under high vacuum and hydrated with HEPES/EDTA buffer solution (5 mM HEPES, 0.1 mM EDTA, pH 7.4) (Sigma-Aldrich, Chemical Company (Co.), St. Louis, MO, USA) to obtain 1.0 mM overall lipid dispersion. The solution was vortex-mixed and then freeze–thawed six times from liquid nitrogen to 40 °C. The dispersion was then extruded (10 times) using a 10 mL extruder (Lipex Biomembranes, Vancouver, BC, Canada) through a 100 nm polycarbonate membrane (Whatman Nuclepore, Toronto, ON, Canada) at 40 °C, well above the transition temperature of DMPC (24.2 °C). The liposomal dispersion was then diluted from 1.0 mM to 200 μM total lipid (gemini = DMPC = 100 nmol/mL) in HEPES/EDTA. A 100 μM siRNA solution was prepared by solubilizing 10 nmol siRNA in 100 μL of HEPES/EDTA and further diluted to 2.6 μM siRNA (2.6 nmol/mL). For the preparation of lipoplexes, 350 μL of siRNA (2.6 nmol/mL) were added to 350 μL of the liposomal dispersion (200 μM total lipid, gemini = DMPC = 100 nmol/mL) to obtain the final lipoplex dispersion at a charge ratio of +2/−1 (final composition: siRNA = 1.3 nmol/mL, gemini = DMPC = 50 nmol/mL). Samples were left to incubate at 25 °C for 8 h before being used for the experiments. In the following days, samples were stored at 4 °C.

#### 2.2.2. Optimization of the Lipid/siRNA Ratio

To understand the effect of a different amount of siRNA on the formation of lipoplexes and on their biological activity, compared to previously published studies where the positive (gemini in liposome)/negative (siRNA) ratio was +2/−1 [11,12,13], here, we also explored ratios such as +3/−1, +1/−1, and +1/−2. Lipoplexes at different charge ratios were prepared by adding 350 μL of siRNA solution (2.6 nmol/mL) to 350 μL of liposomal dispersion at different concentrations (gemini = DMPC = 150 nmol/mL to obtain a +3/−1 final charge ratio, gemini = DMPC = 50 nmol/mL for +1/−1, and gemini = DMPC = 25 nmol/mL for +1/−2, respectively).

#### 2.2.3. Functionalization of Lipoplexes with Siglec-10 Ligand F9

The size, size distribution, ζ-potential, and stability over time of liposomes and lipoplexes were determined by dynamic and dielectrophoretic light scattering measurements (DLS and DELS, Malvern NanoZetaSizer apparatus, Malvern, UK) equipped with a 5 mW HeNe laser equipped with a Peltier system for temperature control. Preparation and characterization are detailed in previously published manuscripts [11,12,13].

Lipoplexes functionalized with the Siglec-10 ligand F9 were prepared using 3 different protocols (I, G, and H) (Supplementary Figure S2). In protocol I (already described in [13]), liposomes functionalized with the F9-Siglec-10 ligand (Sig10L [15]) were prepared according to the above procedure. F9-PEG lipid was added in the film in a 2.8% molar percentage with respect to the overall lipid composition to obtain L2 liposomes. Lipoplexes were prepared starting from L2 liposomes already containing the F9-PEG lipid, as described in the previous paragraph.

In protocol G, after extrusion of the DMPC/gemini liposomes (L1, gemini = DMPC = 100 nmol/mL), F9-PEG lipid was added in half the amount of preparation I and incubated for 30 min (min) at 40 °C to obtain the functionalized liposomes (L1+). Finally, lipoplexes were prepared following the same procedure as described above, adding 350 μL of siRNA solution (2.6 nmol/mL) to 350 μL of liposomal dispersion L1+.

In protocol H, after extrusion of the DMPC/gemini liposomes (L1), siRNA was added and incubated for 1 h at room temperature to obtain lipoplex F. F9-PEG lipid was then added to the lipoplex suspension in an amount that is half that of preparation I and incubated for 30 min at 40 °C to obtain lipoplex H.

In addition, we explored the stability of the lipoplexes over time at 25 °C, from Day 0 (after 8 h of preparation of the lipoplexes) to Day 7, considering the different protocols (I, G, and H) for functionalization. After the first control at D0, samples were stored at 4 °C.

#### 2.2.4. Labeling of Lipoplexes with ATTO740 Fluorescent Dye

For fluorescently labeled lipoplexes to be used in in vitro experiments, ATTO740-DMPC fluorescent lipid (abbreviated here as ATTO740, ATTO-TEC GmbH, Martinshardt, Siegen, Germany) was used, adding it in the film preparation step at a 0.1% molar percentage with respect to the total lipids (to have fluorescently labelled L1, L2, F, I dispersions). Following protocol I, ATTO740 was added in the film preparation step to obtain after hydration a suspension of DMPC/gemini/F9-PEG/ATTO740 fluorescent L2 liposomes, consisting of DMPC = gemini = 100 nmol/mL, F9-PEG-lipid = 5.6 nmol/mL (2.8% of total lipid), and ATTO740 = 0.2 nmol/mL (0.1%).

Fluorescent lipoplexes were obtained following protocol I by adding 350 μL of siRNA solution (2.6 nmol/mL) to 350 μL of liposomal dispersion L2.

### 2.3. Binding Efficiency of siRNA to Liposomes

#### 2.3.1. Electrophoresis

Lipoplexes were analyzed by agarose gel (2%) electrophoresis followed by ethidium bromide staining to visualize bound and free siRNA. The siRNA-binding efficiency to liposomes was assessed by adding 10 μL of lipoplex formulation into the wells. Free siRNA was used as the positive control, while the liposomes were used as the negative control.

Lipoplex formulations were analyzed on Days 0 (8 h after preparation), 1, 3, and 7 following their preparation. Electrophoresis was run for 30 min at 37 °C. A real-time UV transilluminator (Invitrogen, Waltham, MA, USA) visualized siRNA bands. The same protocol was employed to analyze DMPC/gemini/F9-PEG lipid/siRNA and DMPC/gemini/F9-PEG lipid/ATTO740/siRNA lipoplexes.

#### 2.3.2. HPLC Determination

The amount of siRNA in each lipoplex sample was determined by high-pressure liquid performance chromatography (HPLC). For calibration curve construction, a stock solution of siRNA in water (100 µM) was dissolved in a buffer solution at pH 7.4 consisting of 5 mM HEPES, 0.1 mM EDTA to obtain different siRNA concentrations (0.25, 0.5, 1, 2, 4, and 8 µM). For stability measurements, a 4 µM siRNA in 5 mM HEPES, 0.1 mM EDTA, pH 7.4 was incubated for 0.5, 1, 2, 4, 8, 24, and 48 h at 25 °C and 37 °C prior to HPLC injection. Lipoplex formulations were treated with methanol at a 1:3 (*v*/*v*) ratio in order to ensure the rupture of the lipoplex prior to HPLC analysis. The optimized sample/methanol *v*/*v* ratio was assessed by DLS analysis (NanoZetaSizer, Malvern, UK). For HPLC analysis, a Dionex UltiMate 3000 UHPLC (Thermo Fisher, Waltham, MA, USA) system equipped with an automatic injector, column heater, and coupled with a DAD-3000 Diode Array Detector (Thermo Fisher) was employed. A Waters Acquity UPLC BEH C18 1.7 µM (2.1 × 150 mm) column kept at a constant 75 °C was used. The eluents included (A) 0.1 M triethylammonium acetate buffer (pH 7.0) in H_2_O and (B) acetonitrile. Gradient elution: 0–2 min: 5% B; 9 min 45% B; 12 min 45% B; 17 min 95% B; 30 min 95% B. The flow rate was set at 0.3 mL/min. The injection volume was 10 µL, and UV detection was 260 nm. Data were acquired and processed using Chromeleon 7.2 SR5 and GraphPad Prism 8.0. Concentrations of siRNA after lipoplex rupture were found by linear regression analysis of the calibration curve.

## 3. Results

### 3.1. Experiment 1: Definition of the Optimal Lipid/siRNA Ratio

The optimal positive (gemini in liposome)/negative (siRNA) charge ratio, with respect to size, ζ-pot, and sample stability, was estimated in the range +3/−1 and +2/−1, confirming previously published data where the +2/−1 ratio was used for lipoplex preparation [11,12]. The +2/−1 ratio was therefore selected for the subsequent optimization of the lipoplex formulations.

**Figure 1:**
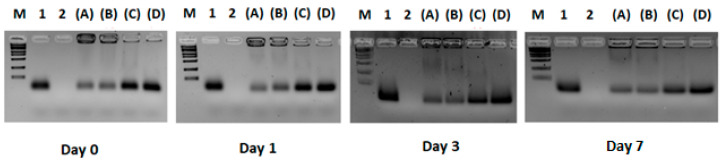
Binding efficiency of siRNA complexed in lipoplexes. Agarose gel electrophoresis/ethidium bromide staining of siRNA complexed in lipoplexes (A–D) with different ratios of lipid/siRNA compared to the migration of free siRNA and liposomes (LP) and a size marker (M). Samples are run for 30 min at 37 °C. M = marker; 1 = siRNA; 2 = liposome (LP); (A) = +3/−1 lipid/siRNA ratio; (B) = +2/−1 lipid/siRNA ratio; (C) = +1/−1 lipid/siRNA ratio; (D) = +1/−2 lipid/siRNA ratio.

As shown in Figure 1, for the +2/−1 and +3/−1 lipid/siRNA ratios, the band corresponding to the free siRNA has a lower intensity with respect to the +1/−1 and +1/−2 lipid/siRNA ratios.

Most importantly, these results indicate that, for the +2/−1 and +3/−1 ratios, the amount of free siRNA in the formulation is negligible compared to the corresponding lipoplex band in the same run, which instead remains locked in the sample well. In addition, the ratio between complexed siRNA and free siRNA for the various formulations seems to remain constant during the time course, thus suggesting the stability of the lipoplexes with respect to the degradation phenomena. The siRNA remains mostly complexed to lipoplexes in wells over a period of 72 h after the preparation.

**Figure 2:**
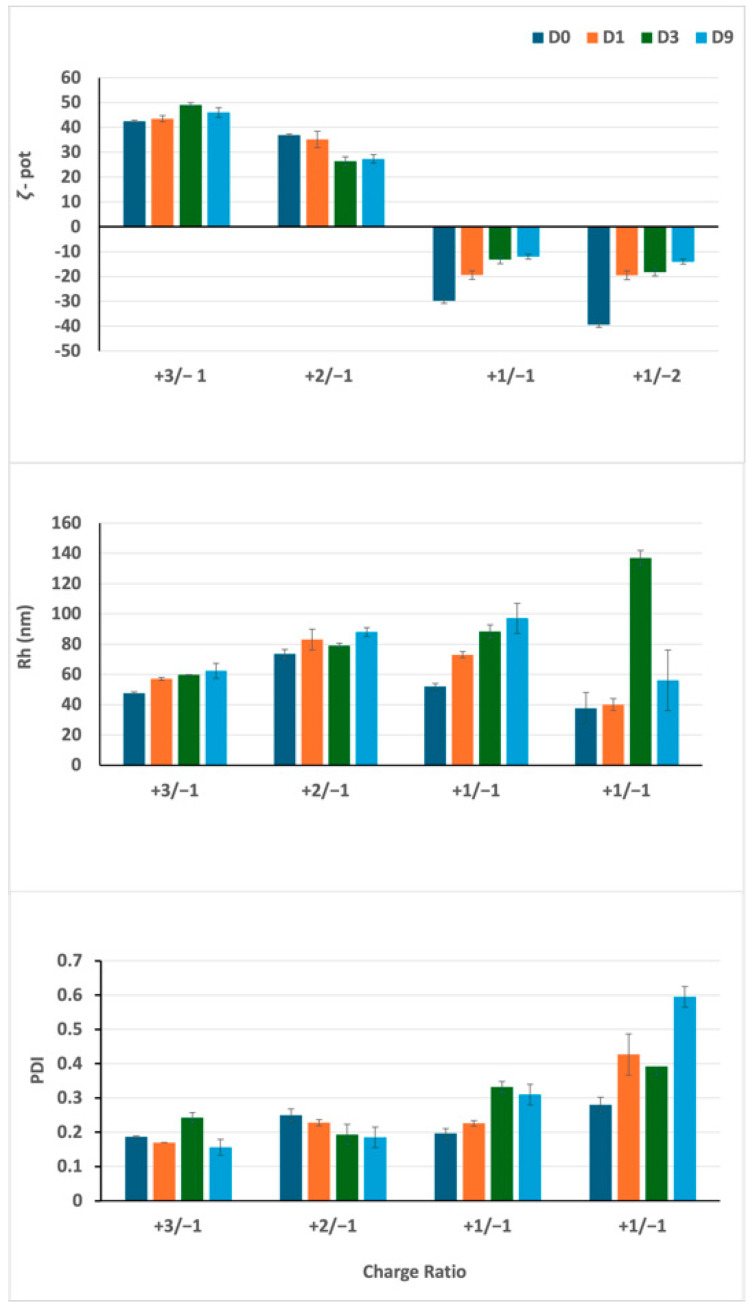
Characterization of lipoplexes as a function of the charge ratio by DLS. The ζ-potential (ζ-pot), the polydispersity index (PDI), and hydrodynamic size (Rh) of the particles are reported over time. Lipoplex dispersions are characterized by the same siRNA concentration (1.3 nmol/mL) and different lipid concentrations (gemini = DMPC = 150 nmol/mL to obtain the +3/−1 final charge ratio, gemini = DMPC = 100 nmol/mL for +2/−1, gemini = DMPC = 50 nmol/mL for +1/−1, and gemini = DMPC = 25 nmol/mL for +1/−2, respectively). Samples analyzed at D0 (8 h after preparation) are reported in dark blue columns, at D1 (24 h) in orange columns, at D3 (72 h) in green columns, and at D9 (216 h) in blue columns.

In Figure 2, DLS analysis revealed that lipoplexes formulated with the +2/−1 ratio, as well as those with the +3/−1 charge ratio, feature a positive ζ-potential. The ζ-potential remains stable over time, as well as particle size and PDI.

For the +1/−1 and +1/−2 charge ratios, a negative ζ-potential was observed as expected, together with a lower stability, as indicated by the trends of Rh, PDI, and ζ-potential.

For these formulations characterized by an excess of siRNA in terms of charge ratio, it is likely that, after an initial distribution of the siRNA on the liposome surface, a progressive reorganization occurs, resulting in an insertion of siRNA into the bilayer structure with a subsequent change in ζ-potential, followed by a reorganization of the lipid matrix.

### 3.2. Experiment 2: Effect of F9-PEG Lipid on Lipoplex Stability

**Figure 3:**
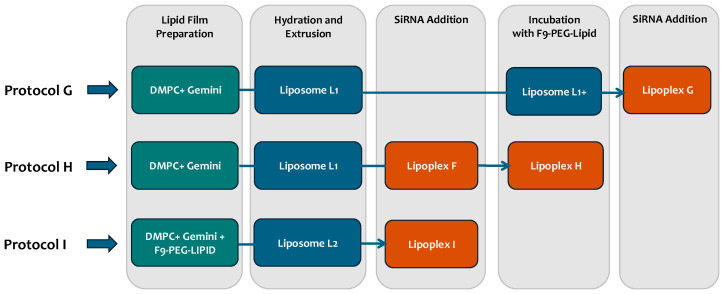
Description of the protocols investigated for the preparation of F9-PEG lipid functionalized lipoplexes.

As already reported, the addition of F9-PEG lipid to the liposome formulation causes an increase in size and PDI [13]. We evaluated the effect of the addition of F9-PEG lipid in lipoplexes as a function of the preparation protocols (Figure 3). In the protocol previously described [13], F9-PEG lipid was added in the film preparation step (here, named protocol I), then we also explored the addition of F9-PEG lipid by incubation soon after liposome preparation (protocol G) and just after lipoplex preparation (protocol H). The best protocol was selected by evaluating the colloidal stability by DLS and DELS measurements and by checking the extent of siRNA complexation by agarose gel electrophoresis.

**Figure 4:**
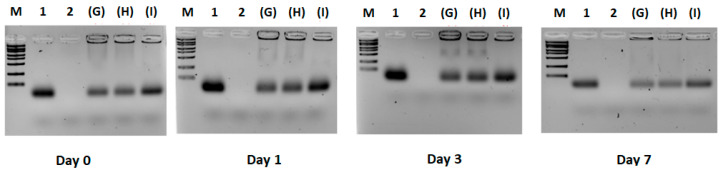
Effect of F9-PEG lipid addition to liposomes. Agarose gel electrophoresis/ethidium bromide staining of siRNA. M = marker; 1 = siRNA; 2 = liposome (LP); (G) = +2/−1 lipid/siRNA ratio; (H) = +2/−1 lipid/siRNA ratio; (I) = +2/−1 lipid/siRNA ratio.

All the three protocols showed a low amount of free siRNA, with the highest amount observed in protocol I (Figure 4). The electrophoresis shows similar results over the time course of the experiment.

**Figure 5:**
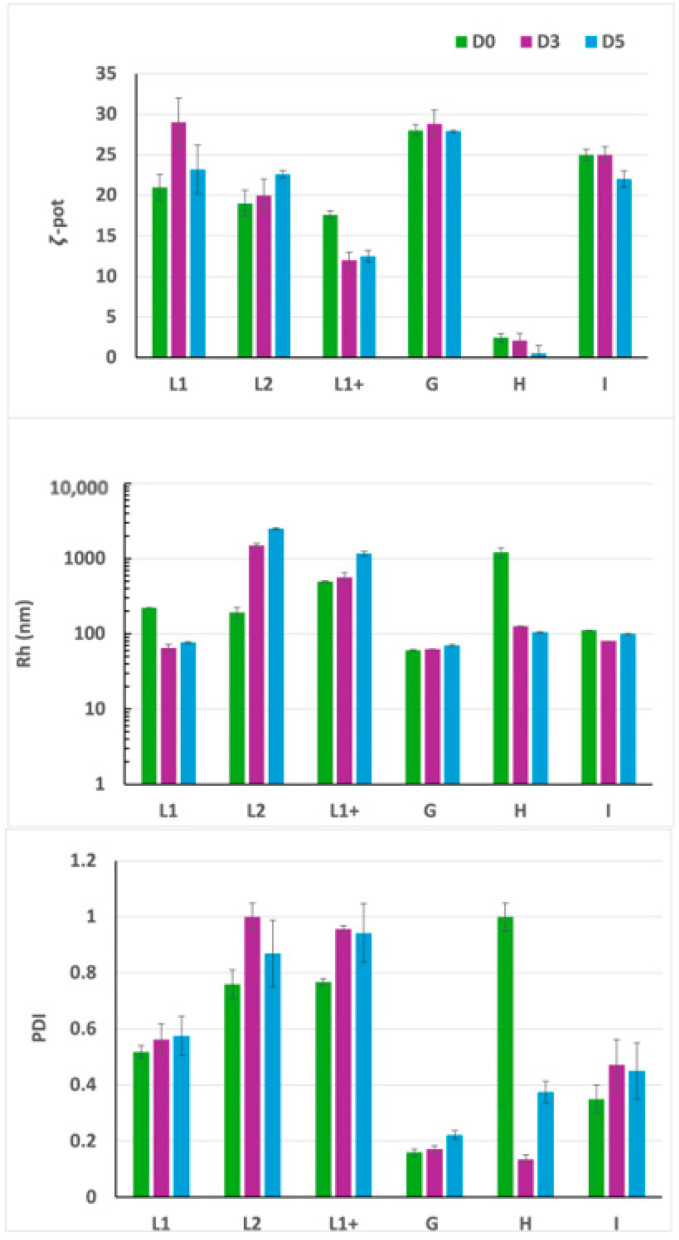
DLS analysis of F9-PEG lipoplexes. F9-PEG functionalized lipoplexes prepared with G, H, and I protocols at the same lipid/siRNA +2/−1 are compared with liposomes L1 (without F9-PEG), L1+ (where F9-PEG is added after extrusion incubation, protocol G), and L2 (where F9-PEG is added in the lipid film before extrusion, protocol I). The ζ-potential (ζ-pot), the polydispersity index (PDI), and hydrodynamic size (Rh) of the particles are reported over time. Samples analyzed at D0 (8 h after preparation) are reported in green columns, at D3 (72 h) in purple columns, and at D5 (120 h) in blue columns.

As evidenced by the DLS analysis, lipoplexes prepared according to protocols I and *G* exhibit a lower PDI (approximately 0.3 ÷ 0.4) in comparison to lipoplexes prepared with protocol H (Figure 5). This finding suggests a higher size homogeneity, along with a reduced tendency towards aggregation, reflecting the presence of more stable lipoplexes for protocols I and G. Furthermore, a lower PDI for formulations prepared with protocols G and I is also accompanied by a significantly higher ζ-potential compared to lipoplexes prepared with protocol H, which should ensure a better interaction with the plasma membrane. The findings of this study demonstrate that protocol H does not result in the formation of lipoplexes that meet the desired criteria, thus leading to its subsequent exclusion from further investigation.

### 3.3. Experiment 3: Effect of ATTO740 on Lipoplex Stability

**Figure 6:**
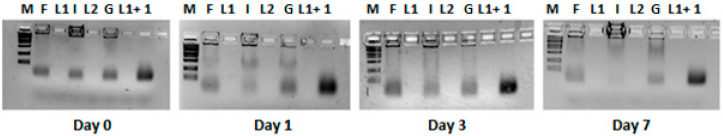
Effect of F9-PEG and ATTO740 addition to lipoplexes. Agarose gel electrophoresis of siRNA in protocols G and I preparations (upper panel) during the established time course of free siRNA compared to siRNA complexed in lipoplexes (bottom panel). The stability of I was confirmed within Day 3. M = marker; F = lipoplexes/ATTO740; L1 = liposome used to prepare F and H; I = lipoplexes/ATTO740/F9-PEG (F9-PEG is added the following extrusion); L2 = liposome used to prepare I; L1+ = liposome used to prepare G; 1 = siRNA.

**Figure 7:**
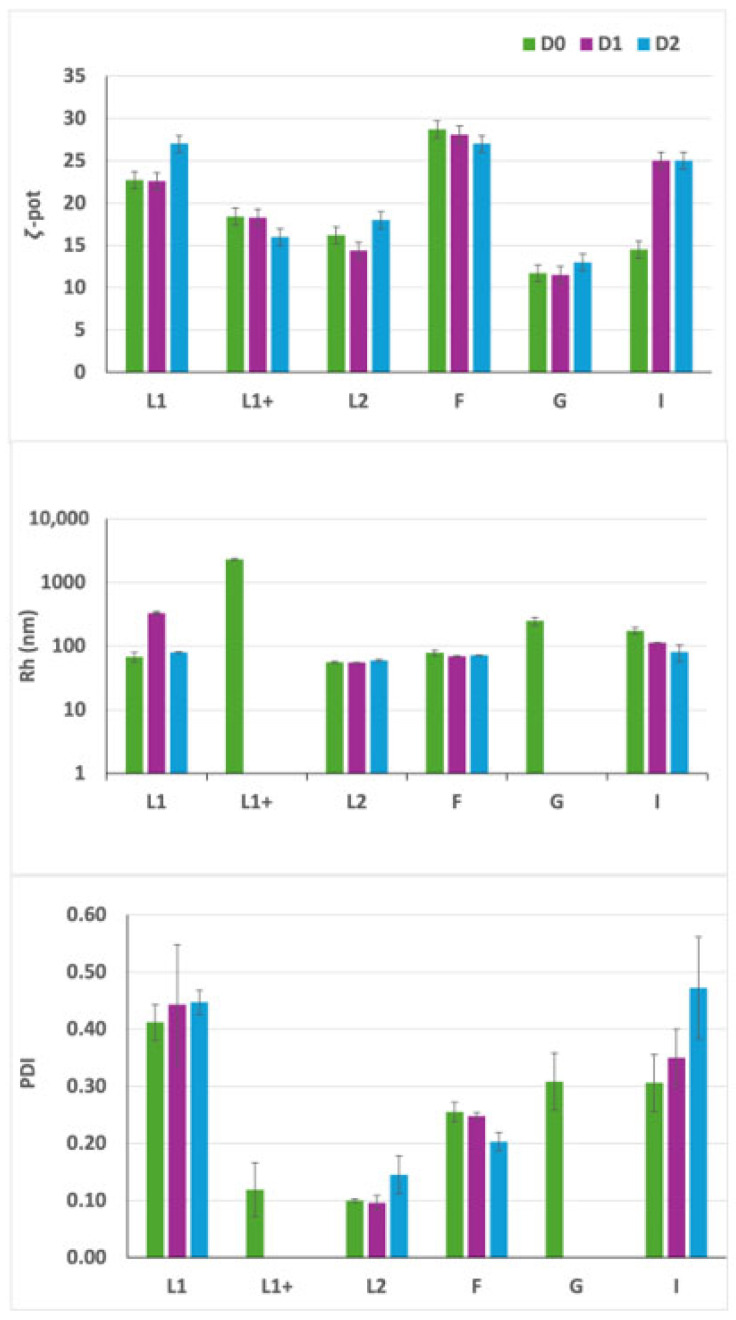
DLS analysis of liposomes and lipoplexes labeled with ATTO740. F9-PEG functionalized dispersions (L1+, L2, G, and I) are compared to not functionalized dispersions (L1 and F). The ζ-potential (ζ-pot), the polydispersity index (PDI), and hydrodynamic size (Rh) of the particles are reported over time. Samples analyzed at D0 (8 h after preparation) are reported in green columns, at D1 (24 h) in purple columns, and at D2 (48 h) in blue columns.

Figure 6 and Figure 7 report the effect of ATTO740 on the two protocols G and I in comparison with the formulation without F9-PEG lipid. As shown in Figure 6, the addition of ATTO740 does not affect siRNA binding to liposomes.

As demonstrated by DELS and DLS experiments, the I preparation exhibits a higher stability over time compared to the G protocol, showing higher ζ-potential and lower diameter over time, despite the relatively high PDI levels (Figure 7). Based on these results, the optimal protocol for preparing functionalized lipoplexes is protocol I, which will then be used for future experiments.

**Figure 8:**
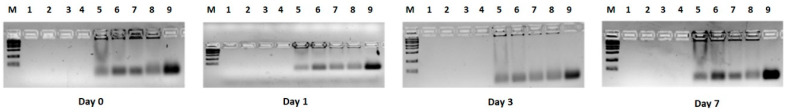
Effect of labeling with ATTO740 on I lipoplexes. Agarose gel electrophoresis of siRNA in the I and F preparations (upper panel) during the established time course of free siRNA compared to siRNA complexed in lipoplexes. In all the different gels are reported: M—Marker; 1—L1: DMPC/gemini [DMPC+gemini] = 4 × 10^−4^ M; [DMPC] = [gemini] = 2 × 10^−4^ M; 2—L1 + ATTO740: DMPC/gemini/ATTO740 [DMPC + gemini] = 4 × 10^−4^ M; [DMPC] = [gemini] = 2 × 10^−4^ M; [ATTO740] = 3.6 × 10^−7^ M; 3—L2: DMPC/gemini/F9-PEG [DMPC + gemini] = 2 × 10^−4^ M; [DMPC] = [gemini] = 1 × 10^−4^ M; [F9-PEG] = 5.6 × 10^−6^ M (3% total lipid, F9-PEG loaded in the film); 4—L2 + ATTO740: DMPC/gemini/F9-PEG/ATTO740 [DMPC + gemini] = 2 × 10^−4^ M; [DMPC] = [gemini] = 1 × 10^−4^ M; [F9-PEG] = 5.6 × 10^−6^ M; [ATTO740] = 1.8 × 10^−7^ M (3% total lipid, F9-PEG loaded in the film); 5—L1 + siRNA; 6—L1 + ATTO740 + siRNA(F); 7—L2 + siRNA; 8—L2 + ATTO740 + siRNA(I); 9—Free siRNA.

**Figure 9:**
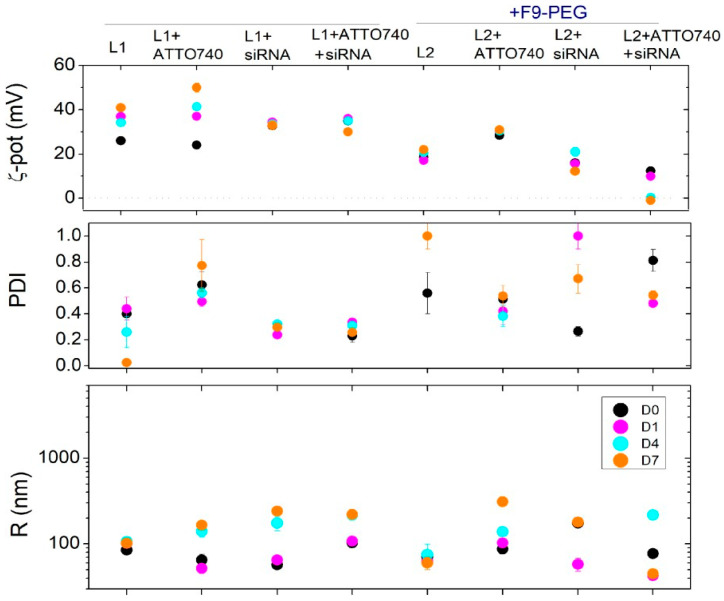
DLS analysis of F9-PEG lipoplex I and labeled with ATTO740. Comparison was carried out with the respective liposomes/lipoplexes formulations not functionalized with F9-PEG and/or not labeled with ATTO740. The ζ-potential (ζ-pot), the polydispersity index (PDI), and hydrodynamic size (Rh) of the particles are reported over time. Samples analyzed at D0 (8 h after preparation) are reported in black circles, at D1 (24 h) in pink circles, at D4 (96 h) in light blue circles, and at D7 (168 h) in orange circles.

In Figure 8 and Figure 9, the effect of ATTO740 in all the formulations (both liposomes and lipoplexes) prepared by the I protocol and those without F9-PEG lipid is compared. Specifically, the effect on stability of the addition of siRNA to the different formulations functionalized with F9-PEG lipid and labeled/unlabeled with ATTO740 fluorescent dye was further sequentially evaluated with respect to the formulations without F9-PEG functionalization (lipoplex F and liposome L1).

As observed in Figure 8, overall, the addition of the ATTO740 dye does not reduce siRNA binding to the liposome in both the F9-PEG functionalized and non-functionalized formulations.

As revealed by DLS analysis (Figure 9), the lipoplexes functionalized with F9-PEG lipid and labeled with ATTO740 exhibit a slightly lower ζ-potential and better properties with respect to siRNA binding and lipoplex formation.

In this case, it is not surprising that the low ζ-pot is not associated with a minor colloidal stability, as usually occurs. In fact, here, the lower ζ-potentials are not associated with a reduction of the overall surface charge of the lipoplexes but are more likely connected to the presence of F9-PEG lipid, which causes an increased distance of the shear plane (where ζ-pot is determined, by definition) from the lipoplex surface, which is reflected in the decrease of measured values. Furthermore, in 24 h, the PDI reduced approximately to values of a monodispersed system, and at the same time, the size decreases, with a tendency to reduce its variability with respect to ATTO740 or the F9-PEG formulations.

In conclusion, the inclusion of ATTO740, on the one hand, leads to the definitive selection of protocol I for the preparation of functionalized lipoplexes and, on the other hand, provides formulations with improved colloidal properties.

### 3.4. HPLC Analyses of Free siRNA and Lipoplex Preparations

The concentration of siRNA in each lipoplex preparation was determined by HPLC analysis under denaturing conditions. After obtaining a calibration curve (y = 7.78x + 0.99, R2 = 0.9981) by analyzing increasing concentrations of siRNA (0.25 to 8 μM) (Figure S2), we tested the stability of free siRNA at 25 °C and 37 °C at increasing incubation times (Figure S3). The peak area corresponding to the siRNA was approximately constant over time, and the formation of additional peaks in the gradient elution was not highlighted, suggesting high stability of siRNA at the investigated temperatures. We then quantified the amount of incorporated siRNA in the L1, L1-ATTO740, L2, and L2-ATTO740 lipoplex preparations. To this end, we diluted each sample with methanol (1:3 *v*/*v*) in order to break the lipoplexes and then verified the effective siRNA release in the external medium by DLS evaluation. We also compared the count rate of each lipoplex sample before and after the 1:3 dilution with either the HEPES/EDTA buffer or methanol.

The stability of siRNA in the presence of ethanol had previously been investigated to rule out its degradation; notably, the samples showed count rate values of ~500 when diluted in 5 mM HEPES, 0.1 mM EDTA at pH 7.4 and values ~10 after dilution with methanol at the same ratio. These results confirm that methanol is capable of solubilizing the lipoplex components and the possibility of using this solution to detect the released siRNA. The HPLC analysis revealed that the concentration of siRNA in all the samples remained at approximately the initial value of 1.3 μM, thereby suggesting that there was no significant loss or degradation of siRNA during lipoplex preparation (Figure S4).

## 4. Discussion

The efficacy of validated and emerging drug molecules has been improved by advanced devices and vehicles such as liposomal carriers being explored for delivery of therapeutic payloads at significant amounts to specific sites.

In order to optimize the lipoplex formulations for the delivery of a novel immunotherapy, we first verified the effect of the charge ratio (lipid positively charged/siRNA negatively charged) on the formation and stability of lipoplexes and confirmed the efficiency of siRNA binding to liposomes by means of electrophoresis, DLS and DELS analyses, and HPLC determination. The ratio +2(gemini)/−1(siRNA) was confirmed as optimal, as in our previous reports [11,12]. DLS and DELS measurements allowed to check lipoplex stability over time. Further, different protocols for functionalization with F9-PEG were explored, confirming that the addition of F9-PEG in the film preparation procedure (protocol I) results in a higher stability of the resulting lipoplex. This protocol also allows siRNA in lipoplex to be promptly available for cellular uptake and internalization. The effect of the addition of ATTO740-DMPC promoted and increased the stability with respect to the F9-PEG lipoplexes, as evidenced by DLS and DELS analyses. HPLC analysis confirmed that free siRNA was stable at 25 °C and 37 °C at increasing incubation times. The concentration of siRNA in all the lipoplex samples remained at approximately the initial value of 1.3 μM, suggesting that there was no significant loss or degradation of siRNA during lipoplex preparation and upon storage. These investigations into the stability of lipoplex formulations functionalized with F9-PEG allowed us to confirm protocol I as the most suitable for their preparation. Having already demonstrated that these formulations are efficiently taken up over time by PBMCs in vitro [20], this study sets the stage for future animal testing of biodistribution with lipoplexes labeled with ATTO740 and carrying murine siRNA, comparing the results obtained with or without F9-PEG functionalization. Further, ATTO740 will allow improved imaging of the biodistribution in PBMCs through the bloodstream by means of IVIS technology and in targeted lymphoid organs [20]. We would expect to visualize a valid uptake, especially in PBMCs, where the siRNA is expected to produce its effect of variant inhibition within the first 24 h from injection. This evaluation is fundamental before proceeding to the injection of functionalized lipoplexes in the NOD mouse model of disease harboring the *Ptpn22* variant R619W equivalent to the human R620W. To this extent, we already provided evidence that siRNA directed against the murine variant in the murine transfected L929 line can efficiently inhibit its expression at the equivalent dose range of inhibition obtained with human siRNA.

## 5. Conclusions

The results of this investigation demonstrate that F9-PEG lipoplexes are versatile nanocarriers for siRNA, offering potential for effective biomolecules selective release to obtain efficient mRNA variant *Ptpn22* suppression. These formulations could be used for a novel personalized immunotherapeutic strategy for the prevention and treatment of endocrine autoimmunity, especially in T1D and APS3v patients harboring the C1858T *PTPN22* variant.

## 6. Patents

Italian Patent 102018000005182 issued on 26 June 2020 and extended as PCT/IT2019/050095, filed on 8 May 2019, in Europe, USA, and China; Inventor: Alessandra Fierabracci MD PhD.

## Data Availability

The original contributions presented in this study are included in the article/Supplementary Materials. Further inquiries can be directed to the corresponding authors.

## Supplementary Materials

The following supporting information can be downloaded at https://www.mdpi.com/article/10.3390/pharmaceutics17060710/s1: Figure S1: Gemini structure; Figure S2: (A) HPLC trace of free siRNA at a 4-μM concentration. (B) Calibration curve correlating the siRNA concentration and the measured area under the curve (AUC) of each HPLC analysis; Figure S3: (A) Evaluation of the stability of free siRNA (4 μM) by HPLC after 0.5–48 h of incubation at 25 °C. (B) Evaluation of the stability of free siRNA (4 μM) by HPLC after 0.5–48 h of incubation at 37 °C; Figure S4: (A) HPLC analysis of the lipoplex preparation (protocol F) following dilution with methanol. (B) HPLC analysis of the F9-PEG lipid lipoplex preparation (protocol I) following dilution with methanol. (C) HPLC analysis of the ATTO740-labeled lipoplex preparation (protocol F) following dilution with methanol. (D) HPLC analysis of the ATTO740-labeled F9-PEG lipid lipoplex preparation (protocol *I*) following dilution with methanol.

## Abbreviations

The following abbreviations are used in this manuscript:

ATD	Autoimmune thyroid disease
T1D	Insulin-dependent diabetes mellitus
APS3v	Polyglandular syndrome Type 3 variant
L-T4	Levo-thyroxine
*PTPN22*	protein tyrosine phosphatase N22 gene
min	minutes
aCD3	Anti-CD3
TCR	T cell antigen receptor
CSK	C-terminal Src kinase
PBMC	Peripheral blood T lymphocytes
PBS	phosphate buffer solution
mAbs	Monoclonal antibodies
TLR	Toll-Like receptor
ASO	Antisense oligonucleotides
RNAi	RNA interference
siRNA	small interfering RNA
SAM	Affinity Siglec-10 sialoside mimetic
DMPC	Dimyristoylphosphatidylcholine
O/N	Overnight
DLS	Dynamic Light Scattering
DELS	Dielectrophoretic Light Scattering
CD	Circular dichroism spectroscopy
ζ-pot	ζ-potential
Sig10L	Siglec-10 ligand
L2	Liposome + F9-PEG-lipid (2.8%)
L1+	Functionalized liposomes, Liposome used to prepare G
L1	DMPC/gemini liposomes, Liposome used to prepare F
F	Lipoplexes/ATTO740
HPLC	High-pressure liquid chromatography
PDI	Polydispersity index
R	Hydrodynamic size
LP	Liposome
M	Marker
