# Correction: Sennato et al. Optimization of Lipoplexes Functionalized with a Sialic Acid Mimetic (F9-PEG) to Target the C1858T *PTPN22* Variant for Preclinical Assessment of a Novel Immunotherapy in Endocrine Autoimmunity. *Pharmaceutics* 2025, *17*, 710

**DOI:** 10.3390/pharmaceutics17121601

**Published:** 2025-12-12

**Authors:** Simona Sennato, Giorgia Paldino, Cecilia Bombelli, Irene Mezzani, Stefania Petrini, Eugenia Belcastro, Domenico Vittorio Delfino, Francesco Fiorentino, Carlotta Marianecci, Alessia Ciogli, Dante Rotili, Francesca Ceccacci, Alessandra Fierabracci

**Affiliations:** 1 CNR-Institute for Complex Systems (ISC), and Physics Department, Sapienza University of Rome, P. le A. Moro 5, 00185 Rome, Italy; simona.sennato@cnr.it; 2 Bambino Gesù Children’s Hospital, Istituto di Ricovero e Cura a Carattere Scientifico (IRCCS), 00146 Rome, Italy; giorgiapld@gmail.com (G.P.); irene.mezzani@opbg.net (I.M.); stefania.petrini@opbg.net (S.P.); eugenia.belcastro@unipi.it (E.B.); 3 CNR-Institute for Biological Systems, Secondary Office of Rome—Sapienza University of Rome, 00185 Rome, Italy; cecilia.bombelli@cnr.it; 4 Department of Medicine and Surgery, University of Perugia, 06129 Perugia, Italy; domenico.delfino@unipg.it; 5 Department of Drug Chemistry and Technologies, Sapienza University of Rome, 00185 Rome, Italy; f.fiorentino@uniroma1.it (F.F.); carlotta.marianecci@uniroma1.it (C.M.); alessia.ciogli@uniroma1.it (A.C.); 6 Department of Science, Roma Tre University, 00146 Rome, Italy; dante.rotili@uniroma3.it

## Addition of an Author

Eugenia Belcastro was not included as an author in the original publication [1]. The corrected authorship and Author Contributions statement appears below. The authors state that the scientific conclusions are unaffected. This correction was approved by the Academic Editor. The original publication has also been updated.

Simona Sennato ^1,†^, Giorgia Paldino ^2,†^, Cecilia Bombelli ^3^, Irene Mezzani ^2^, Stefania Petrini ^2^, Eugenia Belcastro ^2^, Domenico Vittorio Delfino ^4^, Francesco Fiorentino ^5^, Carlotta Marianecci ^5^, Alessia Ciogli ^5^, Dante Rotili ^6^, Francesca Ceccacci ^3,^* and Alessandra Fierabracci ^2,^*

^2^  Bambino Gesù Children’s Hospital, Istituto di Ricovero e Cura a Carattere Scientifico (IRCCS), 00146 Rome, Italy; giorgiapld@gmail.com (G.P.); irene.mezzani@opbg.net (I.M.); stefania.petrini@opbg.net (S.P.); eugenia.belcastro@unipi.it (E.B.)

**Author Contributions:** Conceptualization, F.C., S.S., C.B., D.V.D., D.R. and A.F.; methodology, G.P., F.C., S.P., I.M., S.S., C.B., F.F., E.B. and A.F.; investigation, G.P., F.C., I.M., S.S., C.B., F.F., C.M., A.C., D.R. and A.F.; data curation, F.C., S.P., S.S., C.B., C.M. and A.C.; writing—original draft preparation, F.C., S.S., C.B. and A.F.; writing—review and editing, A.F. and F.C.; supervision, A.F.; project administration, A.F.; funding acquisition, A.F. All authors have read and agreed to the published version of the manuscript.
