# Supplementary material for: Optimization of Lipoplexes Functionalized with a Sialic Acid Mimetic (F9-PEG) to Target the C1858T PTPN22 Variant for Preclinical Assessment of a Novel Immunotherapy in Endocrine Autoimmunity
Source: Pharmaceutics. 2025 May 28;17(6):710. doi: 10.3390/pharmaceutics17060710 (PMC12195924; doi:10.3390/pharmaceutics17060710)
Supplement: Supplementary file 1 [file pharmaceutics-17-00710-s001.zip › pharmaceutics-3549424-supplementary.pdf]

Figure S1:

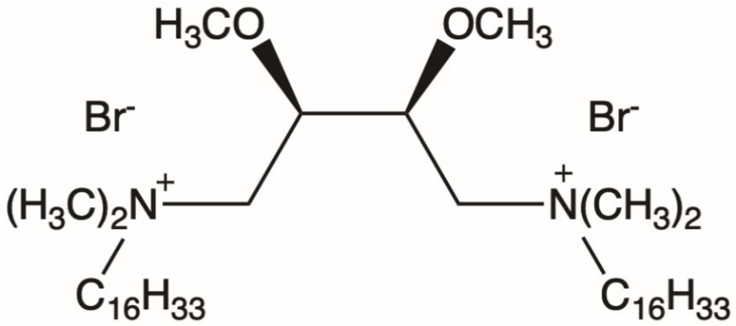

Figure S1: Gemini structure.

Figure S2:

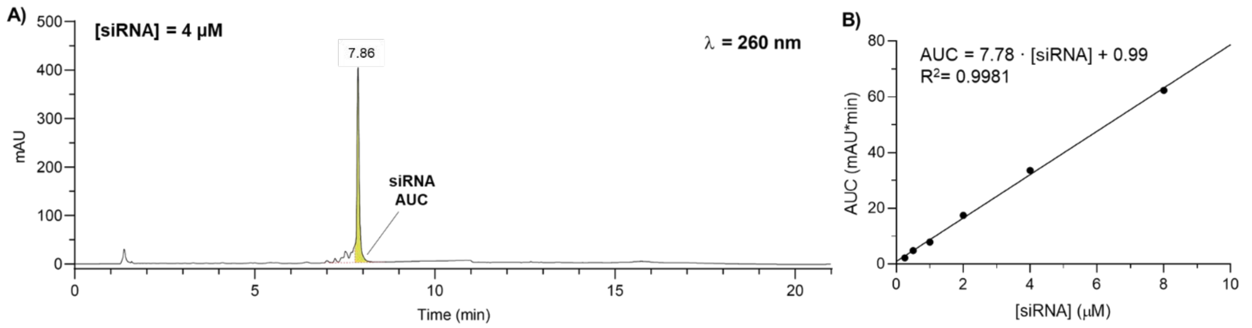

Figure S2: A) HPLC trace of free siRNA at 4 μM concentration**. B)** Calibration curve correlating siRNA concentration and the measured area under the curve (AUC) of each HPLC analysis.

Figure S3:

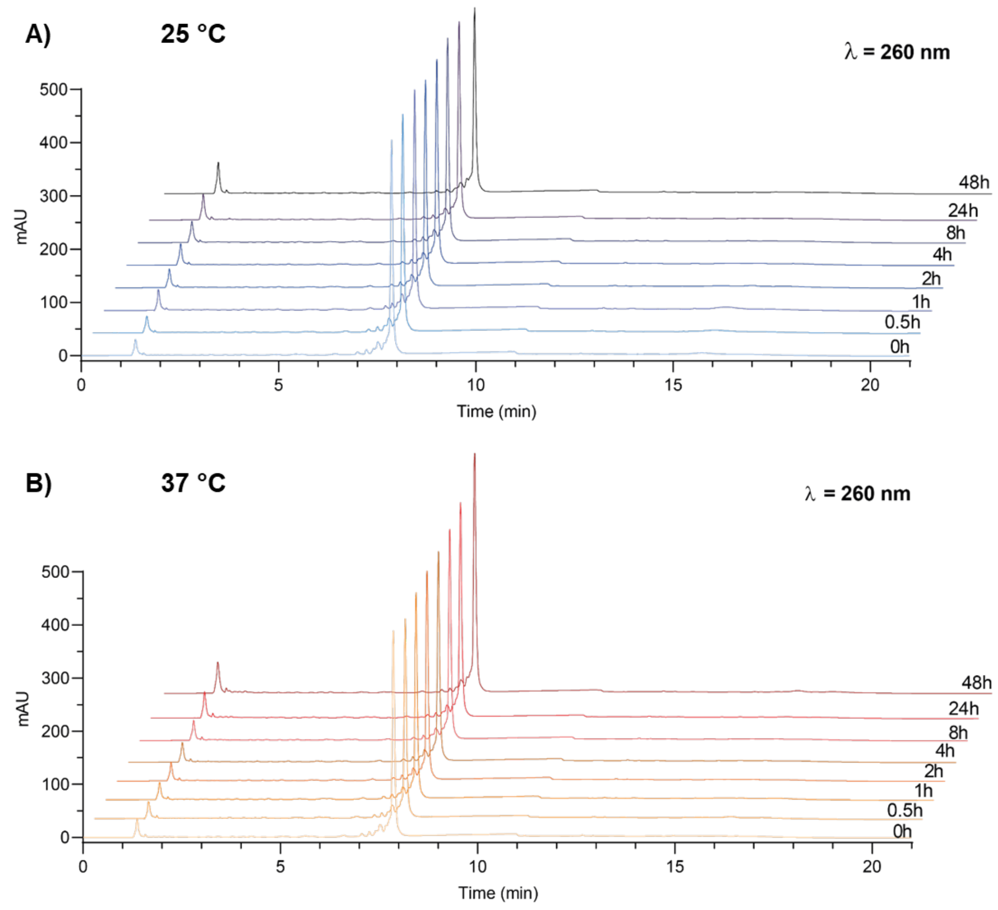

Figure S3: A) Evaluation of the stability of free siRNA (4 μM) by HPLC after 0.5-48 h of incubation at 25 °C. **B)** Evaluation of the stability of free siRNA (4 μM) by HPLC after 0.5-48 h of incubation at 37 °C.

Figure S4.

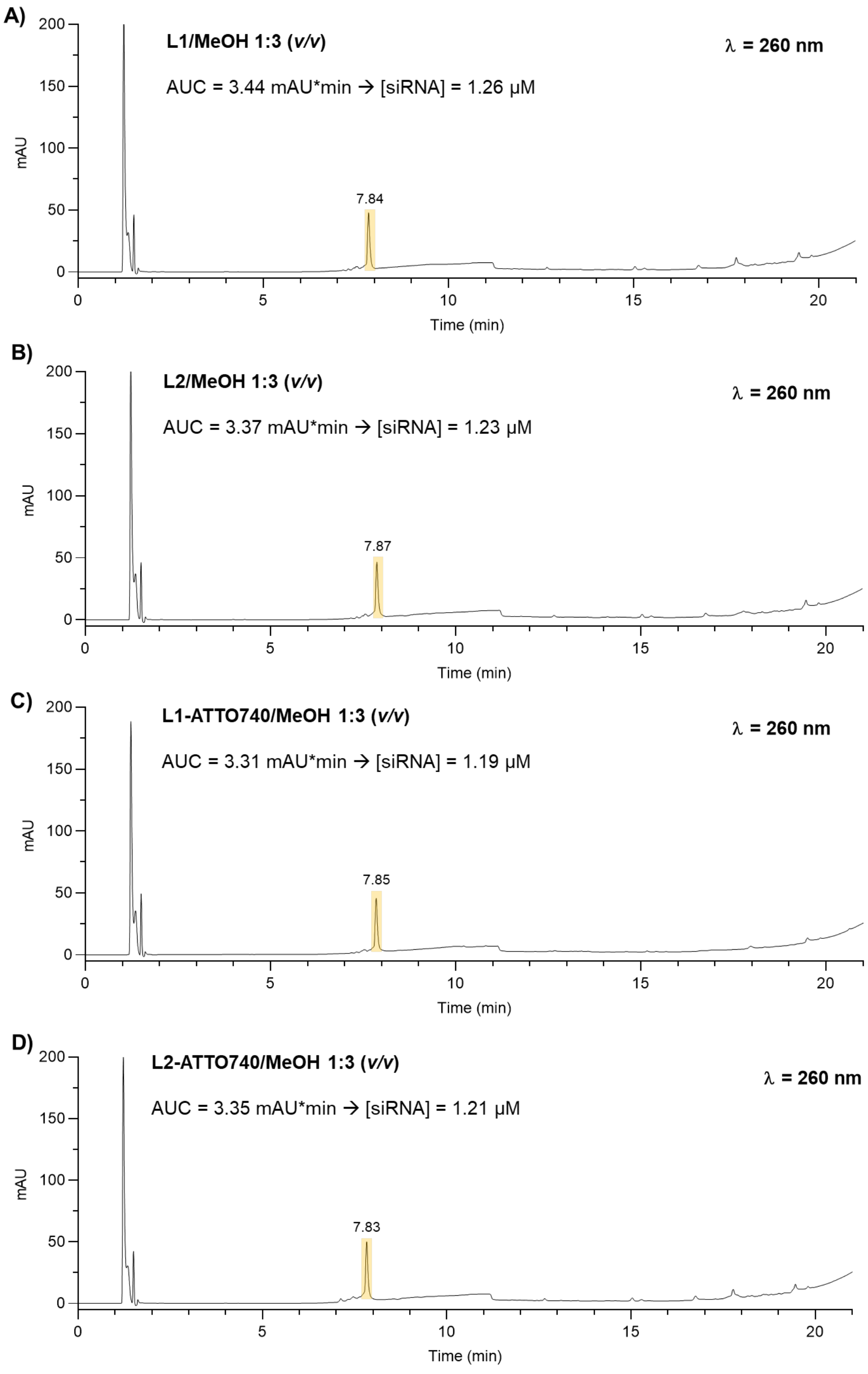

Figure S4*:* **A**) HPLC analysis of the lipoplex preparation (protocol *F*) following dilution with methanol. **B**) HPLC analysis of the F9-PEG-lipid-lipoplex preparation (protocol *I*) following dilution with **C)** HPLC analysis of the ATTO740-labelled lipoplex preparation (protocol *F*) following dilution with methanol. **D)** HPLC analysis of the ATTO740-labelled F9-PEG-lipid-lipoplex preparation (protocol *I*) following dilution with methanol.
